# Livestock Density as Risk Factor for Livestock-associated Methicillin-Resistant *Staphylococcus aureus*, the Netherlands

**DOI:** 10.3201/eid1811.111850

**Published:** 2012-11

**Authors:** Beth J. Feingold, Ellen K. Silbergeld, Frank C. Curriero, Brigite A.G.L. van Cleef, Max E.O.C. Heck, Jan A.J.W. Kluytmans

**Affiliations:** Johns Hopkins Bloomberg School of Public Health, Baltimore, Maryland, USA (B.J. Feingold, E.K. Silbergeld, F.C. Curriero);; National Institute for Public Health and the Environment, Bilthoven, the Netherlands (B.A.G.L. van Cleef, M.E.O.C. Heck);; and Amphia Hospital, Breda, the Netherlands; and VU University Medical Center, Amsterdam, the Netherlands (B.A.G.L. van Cleef, J.A.J.W. Kluytmans)

**Keywords:** swine, livestock, cattle, the Netherlands, geographic information systems, methicillin-resistant *Staphylococcus aureus*, MRSA, cluster analysis, bacteria, zoonoses

## Abstract

To determine whether persons living in areas of high animal density are at increased risk for carrying livestock-associated methicillin-resistant *Staphylococcus aureus* (LA-MRSA), we used an existing dataset of persons in the Netherlands with LA-MRSA carriage and controls who carried other types of MRSA. Results of running univariate and multivariate logistic regression models indicated that living in livestock-dense areas increases the odds of nasal carriage of LA-MRSA. We found that doubling pig, cattle, and veal calf densities per municipality increased the odds of LA-MRSA carriage over carriage of other types of MRSA by 24.7% (95% CI 0.9%–54.2%), 76.9% (95% CI 11.3%–81.3%), and 24.1% (95% CI 5.5%–45.9%), respectively, after adjusting for direct animal contact, living in a rural area, and the probable source of MRSA carriage. Controlling the spread of LA-MRSA thus requires giving attention to community members in animal-dense regions who are unaffiliated with livestock farming.

## Abstract 2

The risk for livestock-associated MRSA increases with increasing density of pigs and calves.

*Staphylococcus aureus* is a zoonotic and human pathogen that can cause a range of health outcomes in humans from minor to life-threatening infections of the skin, bloodstream, respiratory system, urinary tract, and surgical sites (*1*). An increasing proportion of *S. aureus* infections involve drug-resistant strains, including methicillin-resistant *S. aureus* (MRSA) (*2*). In 2007, 171,200 MRSA infections occurred in European Union member states plus Iceland and Norway, resulting in 1,050,000 extra days spent in the hospital (*3*) This translates into excessive hospital inpatient and outpatient costs because of the need to isolate patients and because patients require longer stays and more extensive treatments (*3*). In 2005 in the United States, an estimated 94,000 MRSA infections resulted in >18,000 deaths (*4*).

MRSA has, in the past, been largely associated with hospitals and other healthcare facilities, but since 2000, the majority of MRSA infections in most countries are acquired in the community outside of healthcare settings (*5*,*6*). These strains of community-acquired MRSA are vital public health concerns, but less is known of their origins and routes of transmission. Among these strains of community-acquired MRSA, livestock-associated (LA) strains have been detected in several regions of the world (*7*).

Originally, the LA-MRSA strain studied here was denoted as nontypeable MRSA because of the inability to type it by using standard methods of pulsed-field gel electrophoresis (*8*). It was first detected in the Netherlands in 2003 (*9*) and, as of 2010, accounts for >40% of the MRSA cases in that country (*10*). LA-MRSA has now been identified largely as a single clonal complex on the basis of multilocus sequence typing (ST398), and this clonal complex has a demonstrated association with pigs, cows, and other animals (*11*,*12*), although other clonal complexes have also been shown to be associated with livestock as well. In several European countries, increased risks of carriage have been reported in persons in contact with pigs and veal calves, including farmers, veterinarians, and slaughterhouse workers (*13*,*14*). In the Netherlands, among these occupational groups, the prevalence of ST398 carriage is roughly 42% (*15*), whereas the prevalence of any strain of MRSA in the general population is <1% (*16*).

The emergence and transmission of LA-MRSA among humans and animals (such as poultry, horses, companion animals, pigs, and cattle) have recently been reviewed (*17*). Most epidemiologic studies have focuses on identifying individual and farm level characteristics associated with LA-MRSA carriage and on studying those in direct contact with livestock. In 1 study among pig farmers and their household members, 30% were carriers of MRSA ST398, and the risk for carriage was related to direct exposure to pigs (*15*). A study among veterinarian field workers found that after short-term occupational exposure to pigs, 17% of them carried MRSA. However, >90% of those lost this carriage the next day (*18*). Another study found a clear association between carriage and the duration of contact with veal calves and that carriage was strongly reduced after a period of absence from animal contact (*11*,*19*). These studies, which indicate a high risk for carriage from livestock contact but little persistence of carriage after interruption of animal contact, bring into question the ability of LA-MRSA to spread into the population. Only 1 study examined the role of living in a livestock-dense region as a risk factor as well but did not find it to be a risk factor (*20*). This study used a random mailing in the 3 most pig-dense municipalities in the Netherlands. Of the 534 adult respondents without livestock contact, 1 person was positive for MRSA (0.2%), compared with 13 of 49 persons who worked or lived on a livestock farm (26.5%).

In 2007, risk factors for LA-MRSA carriage in the Netherlands were investigated by van Loo and colleagues (*8*). They found that risk factors for increased odds of LA-MRSA carriage included contact with livestock, acquiring MRSA through known risk factors such as travel to a foreign country or recent interaction with the healthcare environment, and living in a rural area. Our study, conducted during 2008–2011, built upon this work to test the hypothesis that persons living in areas of high pig density may be at increased risk for carrying LA-MRSA. We did this by combining information about where persons lived and what the livestock density was in these areas for which existing information on risk factors had been determined in the 2007 study.

## Methods

### Data

We used data from van Loo et al. (*8*), which consisted of records of all index case-patients with nontypeable MRSA carriage (now referred to as LA-MRSA) from the Netherlands from its emergence in 2003 through September 2005, before the country adopted active surveillance of high risk populations. Information on case-patients and controls was obtained from a national MRSA surveillance program through the country’s Institute for Public Health and the Environment (www.rivm.nl/mrsa). Case-patients were LA-MRSA index patients, that is, the first in a cluster of persons who tested positive for LA-MRSA from a given reference laboratory. Each LA-MRSA case-patient was matched with 2 controls from the same laboratory. The controls were also index patients, but had tested positive for a typeable strain of MRSA (T-MRSA) instead of to LA-MRSA. Further details on subject selection can be found in the original article (*8*).

The same variables used in the 2007 study were used in this current study: contact with pigs, contact with cows, the probable source of MRSA, and whether one lived in a rural area. Probable source of MRSA was placed by the original authors into the following categories: healthcare setting, foreign source (such as travel to another country), other source, or an unknown source (*8*). Our study added municipality level variables of livestock and human population densities and location of residence of study participants. (Municipalities are administrative boundaries in the Netherlands that comprise provinces. In 2005, there were 498 municipalities.) To accomplish this, our inclusion criteria were residence in the Netherlands, availability of information on individual contact with livestock, and geographic information sufficient to support mapping each person to the 6 digit postal code of his or her residence.

We then assigned spatial coordinates to LA-MRSA case-patients and T-MRSA controls on the basis of their 6-digit postal code using the geographic information system software, ArcGIS version 9.3 (*21*). When this method was not sufficient, we used Google Earth (*22*).

We downloaded municipality level statistics of population; land area; and numbers of swine, veal calves, and cows in 2005 from CBS StatLine (*23*). Livestock densities and population density were calculated as the number of animals (pigs, cows, and veal calves) per hectare of land in a municipality. In ArcGIS, we determined in which municipality participants lived, and assigned to them municipality-level characteristics of animal and population densities. We determined counts of case-patients and controls for each of the municipalities using ArcGIS. This study was reviewed and approved by the Institutional Review Board at Johns Hopkins Bloomberg School of Public Health.

### Statistical Analysis

Summary statistics for all relevant variables were reported by using STATA version 10 (*24*). We explored the spatial variation in risk for LA-MRSA—or the concept that the risk or odds of acquiring LA-MRSA varies geographically—using spatial methods available in the R statistical package (*25*). We estimated spatial intensity of case-patients and controls for locations across the study area, defined respectively as the expected numbers of case-patients and controls per km^2^. Spatially varying intensity provides an estimate of regions of high and low densities of case-patients and controls. Spatial intensity is often measured as weighted counts as described (*26*). We used the quartic kernel as our weighting scheme in this study. Using the intensity estimates, we calculated the spatial odds of LA-MRSA to compare the geographic variation of case-patients and controls across the study area. The spatial odds of LA-MRSA per km^2^ compared with those for T-MRSA per km^2^ were calculated as the ratio of estimated case-to-control intensities (*26*).

Contact with pigs, contact with cows, and rural (versus urban) residence were modeled as binary variables. Probable MRSA source was a categorical variable. We compared probable acquisition of MRSA from a foreign country, acquisition from another source, or acquisition from an unknown source with the referent group of healthcare-related acquisition.

We determined goodness of fit of the models using Akaike information criteria and the Hosmer-Lemeshow goodness-of-fit test. Likelihood ratio tests were used to compare multivariate nested models. The densities of livestock were right skewed; thus, we log-transformed the variables to create a more linear relationship between animal density and log odds of LA-MRSA. For ease of interpretation, instead of the 1 log increase in livestock densities, we used a doubling of livestock densities, which is calculated by raising 2 to the power of the β of the density coefficient in the logit model (*27*). Variograms were used to diagnose possible spatial variation in regression residuals, with inference on regression parameters adjusted accordingly.

In a separate but related analysis, we identified specific clusters of LA-MRSA. We used SaTScan version 9.0 (*28*) to conduct this cluster detection analysis with a Poisson model of counts of case-patients per municipality after adjusting for population size as described (*28*). SaTScan is a software package that is used to analyze spatial and temporal patterns in data. It uses a moving window method (in this application, over a set of contiguous municipalities) and determines the presence of a cluster on the basis of whether the estimated risk within a window is significantly greater than the estimated risk outside of the window. Statistical significance is based on the null hypothesis of Poisson constant risk (*28*). We created maps showing the identified clusters after adjustment of population density per municipality in ArcGIS. We made similar maps after further adjusting for pig, cow, and veal calf densities per municipality.

## Results

### Study Population Characteristics

Descriptive statistics of the study population are shown in Table 1. From the total population used in the Van Loo analysis of 111 persons (35 case-patients, 76 controls) (*8*), 87 persons (27 case-patients, 60 controls) were included in our study after we excluded persons who lived outside of the Netherlands (n = 4), persons for whom spatial information was insufficient (n = 3), and persons for whom information about individual contact with livestock was lacking (n = 17).

**Table 1:** Characteristics of population in study of LA-MRSA carriage, the Netherlands, 2003–2005*

Characteristic	MRSA status, no. (%) persons		Total no. (%) persons	p value†
	LA-MRSA	T-MRSA		
Total, N = 87	27	60	87	
Probable source				
Health care setting	3 (11.11)	30 (50.00)	33 (37.93)	
Foreign	2 (7.41)	3 (5.00)	5 (5.75)	
Unknown	13 (48.15)	20 (33.33)	33 (37.93)	
Other	9 (33.33)	7 (11.67)	16 (18.39)	0.001
Contact with livestock and farms				
With pigs	10 (37.04)	3 (5.00)	13 (14.94)	0.000
With cows	7 (25.93)	1 (1.67)	8 (9.20)	0.001
Residential location				
Rural	12 (44.44)	4 (6.67)	16 (18.39)	0.000
Livestock density in municipality, animals/ hectare/ municipality				
Pigs				
Quartile 1, 0.000–0.004	0	16 (26.67)	16 (18.39)	
Quartile 2, 0.005–0.651	5 (18.52)	15 (25.00)	20 (22.99)	
Quartile 3, 0.652–3.268	6 (22.22)	12 (20.00)	18 (20.69)	
Quartile 4, 3.269–45.477	16 (59.26)	17 (28.33)	33 (37.93)	0.003
Cows				
Quartile 1, 0.00–0.340	3 (11.11)	30 (50.00)	33 (37.93)	
Quartile 2, 0.341–0.848	8 (19.63)	8 (13.33)	16 (18.39)	
Quartile 3, 0.849–1.496	3 (11.11)	14 (23.33)	17 (19.54)	
Quartile 4, 1.497–5.920	13 (48.15)	8 (13.33)	21 (24.14)	0.000
Veal calves				
Quartile 1, 0.000–0.000	0	20 (33.33)	20 (22.99)	
Quartile 2, 0.001–0.013	4 (14.81)	13 (21.67)	17 (19.54)	
Quartile 3, 0.014–0.178	10 (37.04)	13 (21.67)	23 (26.44)	
Quartile 4, 0.179–4.818	13 (48.15)	14 (23.33)	27 (31.03)	0.000
Population density				
Quartile 1, 0.250–2.027	11 (40.74)	6 (10.00)	17 (19.54)	
Quartile 2, 2.028–3.649	11 (40.74)	11 (18.33)	22 (25.29)	
Quartile 3, 3.650–9.175	2 (7.41)	18 (30.00)	20 (22.99)	
Quartile 4, 9.176–57.11	3 (11.11)	25 (41.67)	28 (32.18)	0.000

*MRSA, methicillin-resistant *Staphylococcus aureus*; LA-MRSA, livestock associated MRSA; T-MRSA, typeable MRSA. †Fisher exact test for differences in MRSA status by covariate categories.

Of the 87 subjects with complete case information, most of those who had contact with pigs (10/13, 76.9%) and cows (7/8, r 87.5%) were LA-MRSA case-patients. Three subjects had contact with both pigs and cows, 2 of whom were case-patients. Twelve of 27 persons without any direct contact with livestock were LA-MRSA positive (44.4%).

**Figure 1:**
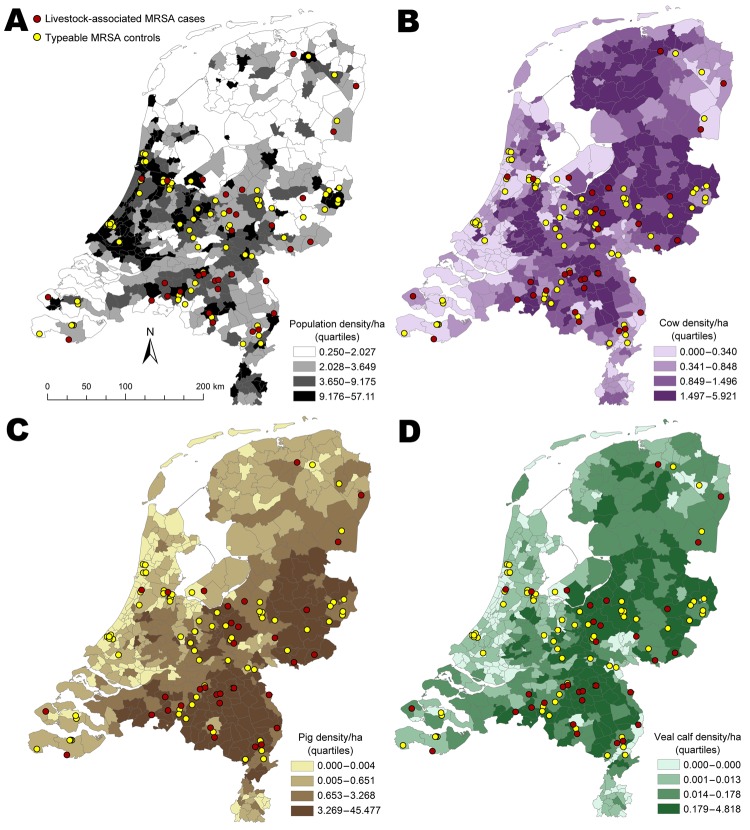
A) Case-patients with livestock-associated methicillin-resistant *Staphylococcus aureus* (LA-MRSA) and controls with typeable MRSA, according to population density, the Netherlands, 2003–2005. B) Density of cattle per municipality. C) Density of pigs per municipality. D) Density of veal calves by municipality.

Specific locations of case-patients and controls are plotted against municipality level population (Figure 1, panel A), cow density (Figure 1, panel B), pig density (Figure 1, panel C) and veal calf density (Figure 1, panel D). Case-patients and controls had significant differences in human and livestock densities per municipality (Table 1).

### Spatial Odds

**Figure 2:**
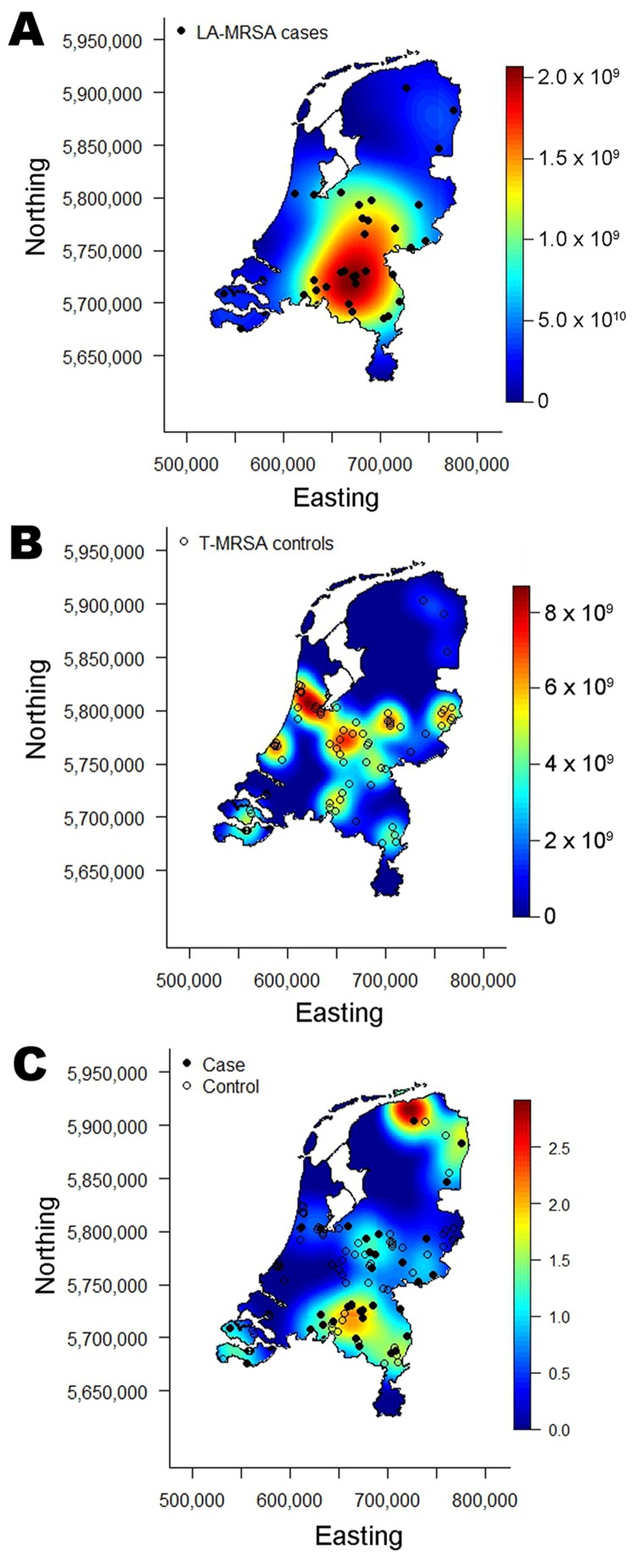
A) Spatial intensity of case-patients with livestock-associated methicillin-resistant *Staphylococcus aureus* (LA-MRSA); B) spatial Intensity of controls with typeable MRSA (T-MRSA); and C) calculated spatial odds for LA-MRSA compared with those for T-MRSA, the Netherlands, 2003–2005.

Relatively high concentrations of controls are seen in general areas of high population density while higher spatial concentrations of case-patients are seen in the more agricultural areas of the country (Figure 2, panels A, B). We estimated spatial odds to give a visual assessment of the spatial variation in risk across the Netherlands (Figure 2, panel C). It is evident that the greatest differences in odds between case-patients and controls are in general areas of high pig density, as was originally reported by van Loo and colleagues (*8*). The elevated spatial odds in the northern part of the country are a spurious result because of small numbers of case-patients and controls and not something that we put forth as a valid result.

### Univariate Logistic Regression

Univariate models results are reported in Table 2. Persons who have contact with pigs have 11.18 times the odds of carrying LA-MRSA (compared with odds of carrying T-MRSA) than persons without pig contact (95% CI 2.76–45.30; p<0.001). A similar relationship is seen when persons with and without contact with cows are compared (odds ratio [OR] 20.65; 95% CI 2.39–178.31; p <0.006). Living in a rural area rather than living in an urban area is associated with 11.2 times the odds for LA-MRSA compared with T-MRSA (95% CI 3.15–39.76; p<0.000). Carriage of MRSA from an unknown or “other” source, as compared to healthcare settings (a priori known to be associated with typeable MRSA), was significantly (p<0.05) associated with the odds of LA-MRSA carriage as compared to T-MRSA.

**Table 2:** Risk factors for LA-MRSA in comparison with those for T-MRSA, the Netherlands, 2003–2005*

Variable	Univariate models		AIC
	OR (95% CI)	p value	
Contact with pigs	11.176 (2.76–45.29)	0.001	97.81
Contact with cows	20.65 (2.39–178.31)	0.006	99.42
Rural	11.20 (3.15–39.76)	0.000	95.21
Probable source of MRSA		0.083	101.02
Foreign vs health care setting	6.67 (0.78–57.06)		
Unknown vs health care setting	6.50 (1.64 25.76)	0.008	
Other vs health care setting	12.86 (2.75–60.22)	0.001	
Livestock density/municipality			
Log (pig)	1.45 (1.13–1.86)	0.003	95.58
Log (cow)	2.25 (1.40–3.60)	0.001	96.32
Log (veal calf)	1.30 (1.11–1.52)	0.001	98.70
Log (population)	0.36 (0.20–0.64)	0.001	94.78

*MRSA, methicillin-resistant *Staphylococcus aureus*; LA-MRSA, livestock-associated MRSA; T-MRSA, typeable MRSA; AIC, Akaike information criteria.

We found that when pig density per hectare is doubled within a municipality, the odds of acquiring LA-MRSA in a univariate model are increased by 29.5% over the odds of acquiring T-MRSA (p<0.003). Similarly, doubling cow and veal calf densities increases the odds of acquiring LA-MRSA compared with those for acquiring T-MRSA by 75.4% (p<0.001) and 19.8% (p<0.001), respectively.

When the inclusion criteria required by our study were used, 16% of original data were lost. We conducted sensitivity analyses by coding all persons with missing livestock contact information as either all having contact or as all not having contact to produce what might be bounds for high and low extremes. In all cases, livestock densities remained significant independent risk factors at the 0.05% level (Table 3).

**Table 3:** Results of univariate logistic regression including missing data on contact with livestock, the Netherlands, 2003–2005*

Contact with livestock	All missing having livestock contact			All missing having no livestock contact	
	OR (95%CI)	p value		OR (95% CI)	p value
Pigs	3.83 (1.56–9.41)	0.003		9.86 (2.49–38.94)	<0.001
Cows	3.20 (1.26–8.13)	0.015		18.85 (2.21–160.68)	<0.007

*OR, odds ratio.

### Multivariate Models

Multivariate model results are reported in Table 4. Model 1 is based only on the original individual level variables from van Loo’s study (*8*): contact with pigs and cows, rural versus urban residence, and information on patient’s probable source of MRSA. In model 1, both contact with pigs and rural residence remain significant predictors as they were in the univariate models, even when adjusting for contact with cows and probable MRSA source. Odds for LA-MRSA compared with those for T-MRSA were 11.6 times higher for a foreign source of MRSA than they were with a healthcare source (95%CI 1.04–129.63; p<0.046). Similarly, the odds were 9.56 when persons with an unknown source were compared with those with a healthcare source (95% CI 1.76–51.93; p<0.009). Acquiring MRSA from another (other) source compared with healthcare acquisition also had increased odds, but this result was not significant (OR 4.3, 95% CI 0.55–33.56; p<0.164).

**Table 4:** Results of multivariate logistic regression for LA-MRSA carriage compared with those for T-MRSA carriage, the Netherlands, 2003–2005*

Variable	Model 1: individual level			Model 2: model 1 + pig density			Model 3: model 1 + cow density			Model 4: model 1 + veal calf density	
	OR (95% CI)	p value		OR (95% CI)	p value		OR (95% CI)	p value		OR (95% CI)	p value
Individual level											
Contact with pigs	8.63 (1.23–60.40)	0.030		6.41 (0.77–53.35)	0.086		6.84 (0.86–54.49)	0.069		9.41 (1.24–71.26)	0.030
Contact with cows	7.37 (0.57–94.68)	0.125		8.39 (0.55–129.18)	0.127		5.10 (0.39–65.87)	0.212		6.18 (0.53–71.83)	0.146
Rural	5.63 (1.02–31.17)	0.048		4.14 (0.64–26.65)	0.135		5.55 (0.89–34.56)	0.066		4.94 (0.802–30.41)	0.085
Probable source of MRSA											
Foreign vs. health care	11.61 (1.04–129.63)	0.046		8.53 (0.72–100.45)	0.088		8.71 (0.74–102.73)	0.086		14.36 (1.06–193.53)	0.045
Unknown vs. health care	9.56 (1.76–51.93)	0.009		11.47 (2.01–65.64)	0.006		14.03 (2.25–87.47)	0.005		13.31 (2.02–87.75)	0.007
Other vs. health care	4.30 (0.55–33.56)	0.164		4.12 (0.54–31.32)	0.032		2.91 (0.36–23.77)	0.319		4.11 (0.51–33.00)	0.184
Municipality level											
Log (pig density)				1.37 (1.01–1.87)	<0.041						
Log (cow density)							2.28 (1.17–4.45)	0.016			
Log (veal calf density)										1.37 (1.08–1.72)	0.009
Regression diagnostics											
AIC	84.76			79.98			79.14			77.90	
Hosmer- Lemeshow†	2.52	0.6407		5.48	0.7050		7.52	<0.4817		6.09	<0.6374
Likelihood ratio test	NA				0.0092			<0.0058			<0.0029

*MRSA, methicillin-resistant Staphylococcus aureus; LA-MRSA, livestock-associated MRSA; T-MRSA, typeable MRSA; OR, odds ratio; AIC, Akaike information criteria; NA, not applicable. †Hosmer-Lemeshow, Hosmer-Lemeshow goodness-of-fit test.

Models 2–4 build on model 1 (the base model) by adding in the logs of pig, cattle, and veal calf densities per municipality, respectively, with the same individual level variables used in model 1 (Table 4). Model 2 builds on model 1 by adding a term for the log of pig density. The odds ratio comparing LA-MRSA to T-MRSA for a 1 log increase in pig density per hectare after adjusting for the individual risk factors (the variables in model 1) for LA-MRSA was 1.37 (95% CI 1.01–1.87, p<0.041). A doubling of the pig density per municipality increases the odds of LA-MRSA carriage compared with T-MRSA carriage by 24.7% after adjustment for individual level risk factors. Model 3 builds on model 1 by incorporating the log of the cow density per municipality. Adjusting for the individual level predictors, a 1 log increase in cow density yields a 2.28 increase in odds for LA-MRSA compared with T-MRSA (95% CI 1.17–4.45, p<0.016). Here, a doubling of cow density in a municipality increased the odds of LA-MRSA compared with T-MRSA by 76.9%. The odds ratio of carrying LA-MRSA compared with those of carrying T-MRSA in model 4 for a 1 log increase in veal calf density after adjustment for individual variables was 1.37 (95% CI 1.08–1.72, p<0.009). Thus, a doubling of the veal calf density per municipality yields a 24.09% increase in the odds of carrying LA-MRSA compared with carrying T-MRSA.

The Hosmer-Lemeshaw goodness-of-fit tests indicate that all models fit the data sufficiently well. The Akaike information criteria and likelihood ratio values for models 2–4 indicate that adding area-level animal density variables improves the original model (model 1). Variograms of residuals from the 4 models did not reveal any substantial spatial variation.

### Cluster Detection

**Figure 3:**
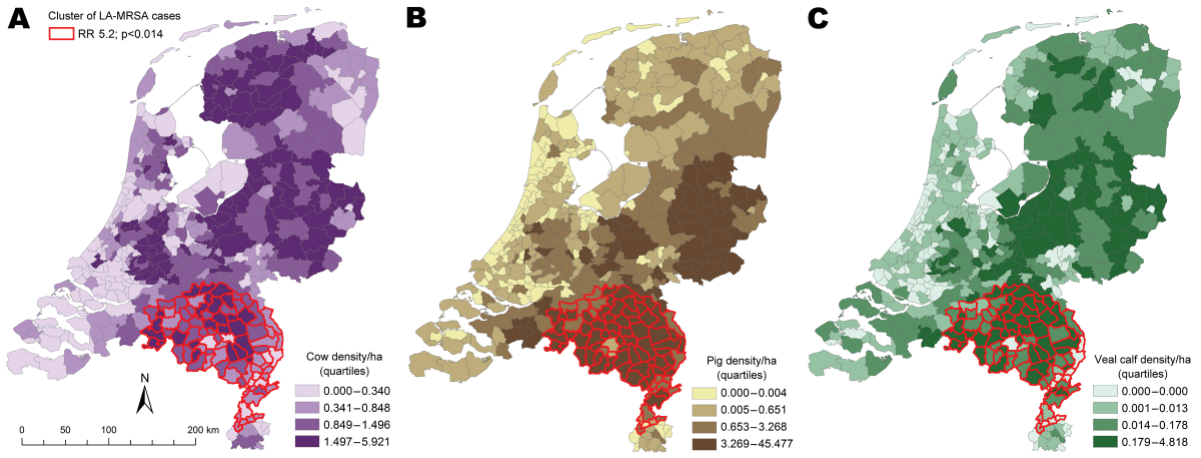
Clusters of livestock-associated methicillin-resistant *Staphylococcus aureus* (LA-MRSA) in the Netherlands, 2003–2005, taking into account 20% population at risk with overlays showing veal calf density (A), cow density (B), and pig density (C). RR, relative risk.

Cluster detection analysis results indicate that after adjusting for the size of the population in a given municipality, 1 significant cluster of LA-MRSA cases (relative risk 5.2, p<0.014) was found when a maximum of 20% of the population at risk was designated as the maximum spatial cluster size. Figure 3 (panels A–C) shows the cluster detection results mapped on top of veal calf density, all cattle density, and pig density for visual identification of associations.

To test whether accounting for livestock density at the municipality level would eliminate the existence of this hot spot of LA-MRSA, we ran additional analyses in SaTScan with adjustment for the density of each animal population separately (*28*). These results indicated that adjusting for animal densities eliminated the presence of the cluster, further supporting the hypothesis that livestock densities per municipality are key risk factors for LA-MRSA carriage.

## Discussion

Our findings indicate that regional density of livestock is a notable risk factor for nasal carriage of LA-MRSA for persons with and without direct contact with livestock. This finding has been emphasized in recent research that found LA-MRSA carriage in persons without connections to the farm environment (*29*). A recent study indicated that proximity to farms is a potential risk factor, even in absence of direct contact between humans and animals (*30*). In addition, MRSA has been found in meat; diet may therefore provide another route of exposure for the general population (*31*,*32*).

We observed in the multivariate analysis that living in a region with high cattle density conferred higher odds of LA-MRSA carriage than did living in a region of high pig or veal calf density. We are not certain what may explain this association, but it does warrant further investigation. We found in our multivariate models that some of the risk factors previously identified by univariate analysis by van Loo and colleagues (*8*) dropped out as being less significant when regional livestock density was included in a multivariate model, such as direct contact with pigs and cows and living in a rural location. Intriguingly, acquiring MRSA from an “unknown” source remained highly significant in all of the multivariate models. These results highlight the value of considering these individual-level variables, together with regional level data, as an update of the univariate analysis conducted by van Loo and colleagues in 2007.

This analysis is limited by the small size of the dataset. However, even with such a small dataset, and after adjusting for known and supposed LA-MRSA risk factors, the densities of livestock per municipality remain strong and independent risk factors for LA-MRSA carriage.

A second limitation of the study is that the case-patients were initially restricted to index case-patients, which inherently selected against detecting secondary transmission. Conducting a future study that includes non–index case-patients would produce a more accurate picture.

A final limitation is the possibility of recall bias in the participants’ reports of exposures to livestock, leading to a misclassification of exposure. Such a nondifferential information bias may have biased our results toward the null hypothesis.

This work has potential policy implications for MRSA surveillance in countries where a substantial percentage of total MRSA cases are LA-MRSA, such as the Netherlands. Starting in 2006, health policy in the Netherlands has required testing for MRSA carriage on admission to the hospital for persons living or working on pig farms. This study suggests that this screening program may need to be expanded to include other persons from municipalities where livestock densities are high.

Although research has indicated that LA-MRSA is not readily transmitted from person to person (*20*,*33*), cases continue to be reported with no identified livestock-associated risk factors. Some possible modes of exposure could involve contact with other domesticated animals, person-to-person contact, and contact with contaminated meat or, in some cases, environmental pathways such as air or waste releases from farms to the surrounding community. Future research should assess these factors in terms of their relationship to living in livestock-dense areas and the likelihood of exposure to MRSA with a larger sample sizes. Information from the statistically significant cluster in the cluster detection analysis can be used to target interventions in the Netherlands. Future work should investigate more recent cases, specifically those without direct links to livestock farming.

We confirm what has been suggested in other studies that veal calf farming (not just pig farming) is a risk factor for LA-MRSA. We also demonstrate a relationship between nontypeable MRSA and all cattle, not just veal calves. The hypothesis that a relationship exists between other types of cattle farming and LA-MRSA carriage should also be explored in further research.

These findings also have the potential to affect countries beyond the Netherlands. Although pig farming is an important industry in the Netherlands, its scale there is greatly overshadowed by the density of pig-farming operations in the United States. In the United States, in 2007, there were 75,442 pig farms, 8,206 of which have >2,000 pigs on them (10.9%) (*34*). For comparison, in the Netherlands in 2000, of the 14,524 pig farms, only 983 housed >2,000 swine (6.8%). Future work could investigate the relationship between these more intensive livestock operations and drug-resistant microorganisms, especially LA-MRSA, which at present has not been widely detected in the United States. These research findings will be useful for generating hypotheses regarding the epidemiology of LA-MRSA in the Netherlands and can provide a warning that where one lives may play a critical role in one’s risk of disease.
